# Construct Validity and Test–Retest Reliability of a Free Mobile Application to Evaluate Aerobic Capacity and Endurance in Post-COVID-19 Syndrome Patients—A Pilot Study

**DOI:** 10.3390/jcm12010131

**Published:** 2022-12-24

**Authors:** Roberto Cano-de-la-Cuerda, Carmen Jiménez-Antona, Alberto Melián-Ortiz, Alberto Molero-Sánchez, Ángel Gil-de Miguel, Ángel Lizcano-Álvarez, Valentín Hernández-Barrera, David Varillas-Delgado, Sofía Laguarta-Val

**Affiliations:** 1 Department of Physical Therapy, Occupational Therapy, Rehabilitation and Physical Medicine, Faculty of Health Sciences, Universidad Rey Juan Carlos (URJC), Alcorcón, 28922 Madrid, Spain; 2 Movement Analysis Laboratoy (LAMBECOM), Faculty of Health Sciences, Universidad Rey Juan Carlos (URJC), Alcorcón, 28922 Madrid, Spain; 3 Faculty of Nursing and Physiotherapy, Universidad Pontificia de Salamanca, 28040 Madrid, Spain; 4 Medical Specialties and Public Health, Faculty of Health Sciences, Universidad Rey Juan Carlos (URJC), Alcorcón, 28922 Madrid, Spain; 5 Department of Nursing and Stomatology, Faculty of Health Sciences, Universidad Rey Juan Carlos (URJC), Alcorcón, 28922 Madrid, Spain; 6 Faculty of Health Sciences, Universidad Francisco de Vitoria (UFV), Pozuelo de Alarcón, 28223 Madrid, Spain

**Keywords:** post-COVID-19 syndrome, mobile applications, performance capacity, aerobic capacity, endurance, fatigue, quality of life

## Abstract

Introduction: Disability associated with the symptoms of post-COVID-19 syndrome is one of its main features and can have a considerable impact on care and rehabilitation units. This, linked to a decreased aerobic capacity and endurance in post-COVID-19 syndrome patients, increases interest in studying the potential of mobile applications to assess performance capacity. The purpose of this research was to study how a free mobile application assesses aerobic capacity and endurance and its relationship with aerobic capacity, test-retest reliability, and endurance evaluated by a conventional test, along with fatigue and health-related quality of life. Methods: An observational study was conducted. RUNZI^®^, a free mobile application, was used by mounting a Samsung Galaxy S8 smartphone using a strap on the right forearm while all participants simultaneously performed a 6-Minute Walking Test (6MWT). Construct validity between the 6MWT and the total distance performed evaluated by RUNZI^®^ was explored. Additionally, evaluation scales to assess fatigue (MFIS) and health-related quality of life (SF-36) were used to analyze the construct validity of RUNZI^®^. For test–retest reliability of the app, the same instructions about the 6MWT and procedure with the app were given to all participants at two different time periods. Results: A total of 16 post-COVID-19 syndrome patients (15 females and 1 male) completed the procedure. Distance measured with the RUNZI^®^ showed an excellent correlation with the 6MWT assessed conventionally (*p* < 0.0001; r = 0.851). No statistical correlations were found between the distance assessed by the RUNZI^®^ app with MFIS and the SF-36. Test–retest reliability was found to be close to statistical significance (*p* = 0.058) for distance (m) measured by RUNZI^®^ with an ICC of 0.482. Conclusions: Instrumental 6MWT assessed by the RUNZI^®^ app for the Android^®^ operating system showed an excellent correlation with conventional 6MWT, indicating its construct validity in post-COVID-19 syndrome patients. Further, values for the test–retest reliability for the free mobile application were close to statistical significance with a reliability considered poor in an indoor setting.

## 1. Introduction

Severe acute respiratory syndrome coronavirus 2 (SARS-CoV-2) is the agent that causes coronavirus disease 2019 (COVID-19) [1]. In 2020, the UK’s National Institute for Health and Care Excellence (NICE) published guidance on the long-term consequences of COVID-19, and distinguishes between acute COVID-19, ongoing symptomatic COVID-19, and post-COVID-19 syndrome.

Post-COVID-19 syndrome is a set of signs and symptoms that develop during or after an infection compatible with COVID-19, that continue for more than 12 weeks, and cannot be explained by an alternative diagnosis. Symptoms can often overlap with each other, fluctuate, and change over time, sometimes in the form of relapses. Any body system (cardiovascular, respiratory, gastrointestinal, neurological, musculoskeletal, metabolic, renal, dermatological, and hematological) can be affected. Further, psychiatric problems, generalized pain, fatigue, and persistent fever can be present [2].

It has been highlighted that people who suffered from COVID-19 infection presented impaired physical fitness on discharge from the hospital [3]. In addition, cardio-respiratory impairments may persist for months after discharge. However, post-COVID-19 syndrome patients refer to a fluctuating aerobic capacity and endurance linked to functional mobility and performance of daily life activities [3].

Recent years have seen an increase in the use of information and communication technology (ICT) in healthcare, commonly known as eHealth. The mass adoption of smartphones raises the question of their usefulness as a clinical tool (mHealth). Mobile applications are a promising tool in healthcare as they provide new perspectives for patients and health professionals alike [3]. Mobile apps are programs developed to run on mobile devices, and they are normally adapted to the device’s processor specifications and storage capacity. The purpose of an app is to assist in achieving a specific goal or aid in daily activities. Usually, the interaction between the app and the user is touch-based and apps have been studied for their possibilities in many diseases [4,5,6,7,8], even for specific aims in post-COVID-19 syndrome [9].

In this context, disability associated with post-COVID-19 syndrome is one of its main features and it presents a considerable impact on rehabilitation units [1]. This, linked to a decreased aerobic capacity and endurance in post-COVID-19 syndrome patients, increases the interest in studying the potential of mobile applications to assess performance capacity. Additionally, the mass adoption of smartphones raises the question of their usefulness and validation as a clinical tool [9]. These technological devices must be assessed in terms of clinical feasibility, their psychometric properties as construct validity (established as the relationship between the measure of interest and other related measures), and test–retest reliability.

The purpose of this research was to study the aerobic capacity and endurance assessed by a free mobile application called RUNZI^®^ for the Android^®^ operating system and its relationship with aerobic capacity and endurance evaluated by a conventional test, along with fatigue and health-related quality of life. In addition, test–retest reliability for the app was explored for aerobic capacity and endurance in people with post-COVID-19 syndrome.

## 2. Methods

### 2.1. Design

An observational study was conducted. Strengthening the Reporting of Observational Studies in Epidemiology (STROBE) guidelines were followed to standardize the reporting of this work. This study followed the Helsinki declaration and was approved by the local ethical committee in Madrid (reference number: 21/175). Informed consent was obtained from all the participants.

### 2.2. Participants

Sixteen post-COVID-19 syndrome patients participated in the study with a non-probability sampling. All participants were recruited consecutively from the post-COVID-19 Syndrome Association (Madrid, Spain) between March 2022 and April 2022, with a diagnosis of post-COVID-19 syndrome according to international guidelines [10] by a medical doctor.

The inclusion criteria were as follows: age between 18 and 65 years; a diagnosis of post-COVID-19 syndrome following NICE guidelines; all patients were able to walk independently without aids; no history of orthopedic or arthritic diseases that affected their trunk and lower limb movements; and an appropriate cognitive status to follow simple commands linked to the test performance (≥24 points in the Mini-Mental Test). The exclusion criteria included: a diagnosis of a neurological illness or musculoskeletal disorder; a diagnosis of a cardiovascular, respiratory, metabolic illness, or other conditions which may interfere with the study; and participants had no other diagnosis that could disturb gait ability or visual disturbances noncorrected by optical devices.

All assessments were performed by the same investigator in a controlled setting at the same time of the day. Aerobic capacity and endurance (6-minute walk test, 6MWT), fatigue (Modified Fatigue Impact Scale, MFIS) and quality of life (Short Form-36 Health Survey, SF-36) were assessed for all participants recruited.

### 2.3. Procedure

**Figure 1:**
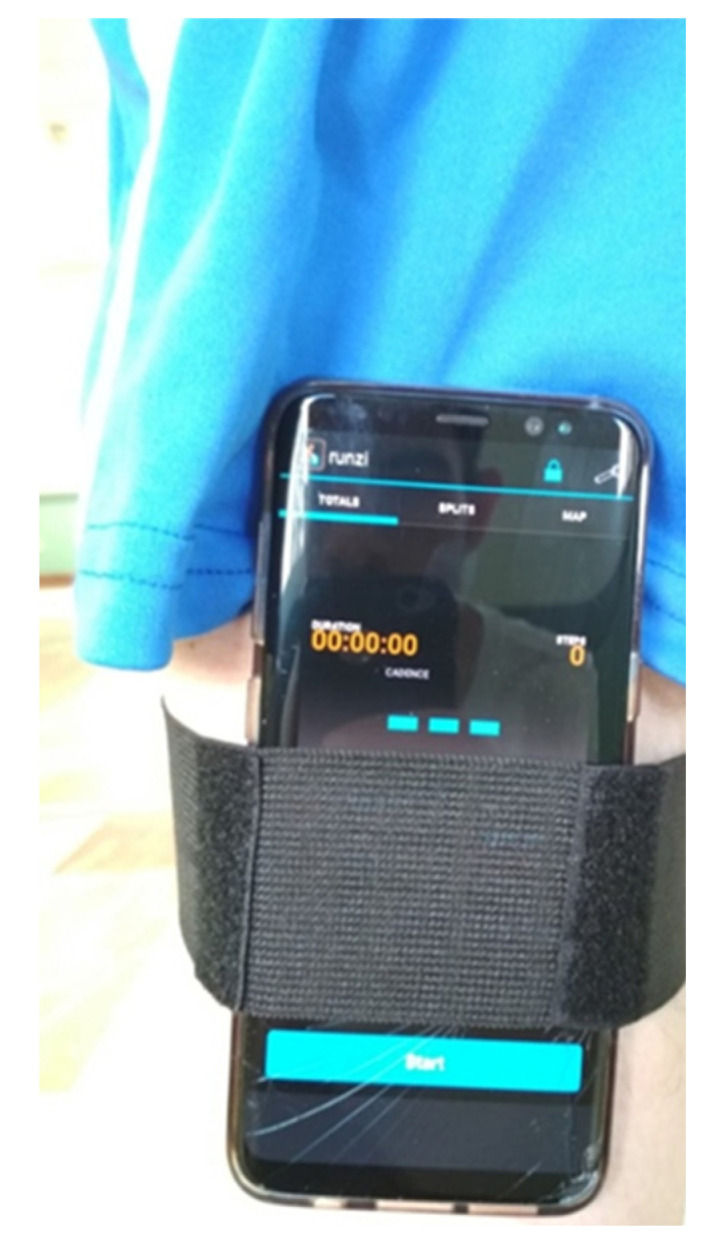
Smartphone mounted on the forearm of the patient.

The RUNZI^®^ (Liketivist developer), free mobile application was used by mounting a Samsung Galaxy S8 smartphone (Samsung Electronics Co., Ltd., Suwon, Republic of Korea) by a strap on the right forearm while all participants simultaneously performed a 6-Minute Walking Test (6MWT) (Figure 1). The app uses the Global Positioning System (GPS) and accelerometer data from the phone to measure gait parameters, such as cadence, time, distance, number of steps, and walking speed.

Prior to the assessment with the app, the procedure was explained to all patients. For this study, only the distance performed (in meters) provided by the app was used for analysis.

#### 2.3.1. Construct Validity

Construct validity between 6MWT (in terms of total distance performed), assessed following the conventional test, and the total distance performed evaluated by RUNZI^®^ was explored. Additionally, evaluation scales to assess fatigue (MFIS) and health-related quality of life (SF-36) were used to analyze the construct validity with RUNZI^®^. Our initial research hypotheses were to obtain a correlation between the 6MWT conventional test and the app, along with expecting to obtain a correlation between fatigue perceived and health-related quality of life with RUNZI^®^.

The 6MWT is a sub-maximal exercise test used to assess aerobic capacity and endurance. The distance covered over a time of 6 min was used as the outcome by which to compare changes in performance capacity [11,12]. To the best of our knowledge, there are no prior studies that have investigated the validity of a mobile application to assess aerobic capacity and endurance in COVID-19 patients. The 6MWT evaluates the functional capacity of an individual and it provides valuable information regarding all the body’s systems during physical activity.

For this study, a 90 m stretch of unimpeded walkway was used. Two cones at either end of the 90 m stretch served as turning points. All patients received the same instructions: “The object of this test is to walk as far as possible for 6 min. You will walk back and forth in this hallway. Six minutes is a long time to walk, so you will be exerting yourself. You will probably get out of breath or become exhausted. You are permitted to slow down, to stop, and to rest as necessary. You may lean against the wall while resting, but resume walking as soon as you are able. You will be walking back and forth around the cones. You should pivot briskly around the cones and continue back the other way without hesitation”. A pulse oximeter for evaluating heart rate and SpO2 was used pre- and post-test. In addition, a stopwatch was used to time the assessment. The total distance performed in meters was recorded as the main result of the 6MWT.

Concurrently, while patients were performing the 6MWT, the RUNZI^®^ free mobile application was used by mounting a Samsung Galaxy S8 smartphone by a strap on the right forearm of all participants. The same rater manually handled RUNZI^®^ to start the data collection synchronized with the beginning of the 6MWT triggered by the stopwatch. The total distance in meters was downloaded from RUNZI^®^ to assess construct validity with the conventional 6MWT.

Prior to aerobic capacity and endurance assessment, MFIS and SF-36 were administered to assess their construct validity and to avoid any interference.

MFIS is a structured, self-report questionnaire modified form of the Fatigue Impact Scale. This instrument provides an assessment of the effects of fatigue in terms of physical, cognitive, and psychosocial functioning. Individual subscale scores can be generated by calculating the sum of specific sets of items. The MFIS consists of 21 items and their administration time is approximately 5–10 min [13].

The SF-36 was used to evaluate health-related quality of life in the sample. It is composed of 35 items, divided into 8 areas: physical function, physical role, emotional role, social function, mental health, general health, body pain, and vitality. It also contains an additional item that is not part of any dimension, and which measures the declared evolution of health. The Spanish version was used for this study [14,15].

#### 2.3.2. Test–Retest Reliability

For the test–retest reliability of the app, the same instructions about the 6MWT and procedure with the app were given to all participants, at two different time periods, following other research on test–retest reliability [16] specifically conducted with the RUNZI^®^ app [17]. The same evaluator performed the test under identical conditions at the same time of the day.

### 2.4. Statistical Analysis

The data were analyzed using version 25.0^®^ of SPSS (Chicago, IL, USA, version 27.0) for Windows software. Pearson correlation coefficients investigated the relationship between the RUNZI^®^ (distance) and the 6MWT, MFIS, and SF-36. Correlation coefficients of 0.00 to 0.49 were interpreted as poor, those of 0.50 to 0.79 as moderate, and those of 0.80 or higher as excellent [18].

The test–retest reliability of the RUNZI^®^ app was assessed using the intra-class correlation coefficient (ICC) and its 95% confidence interval, with a mixed-effect model, consistency (ICC 3,1). The ICC was calculated for the distance performed (meters). Values of the ICC > 0.9 were considered excellent, values of 0.76–0.9 were considered good, values of 0.5–0.75 were considered moderate, and values lower than 0.50 were considered poor [19]. A statistical significance of *p* < 0.05 was established for test–retest reliability.

Finally, the minimal detectable change (MDC) was calculated for test–retest reliability using the following formula: MDC95 = 1.96∗√2∗SEM (where SEM is the standard error of measurement) [20]. To calculate MDC independent of the units of measurement, the MDC% was defined as (MDC/$\bar{X}$)* 100, where $\bar{X}$ is the mean for all observations from test sessions 1 and 2 [21].

## 3. Results

A total of 16 post-COVID-19 syndrome patients (15 females and 1 male) completed the procedure. The mean age of the sample was 46.06 ± 7.92. Table 1 shows the sociodemographic characteristics of the sample. No patient reported discomfort related to carrying the mobile phone on the arm during the test.

The mean distance obtained by conventional 6MWT and assessed by the RUNZI^®^ app are shown in Table 1.

**Figure 2:**
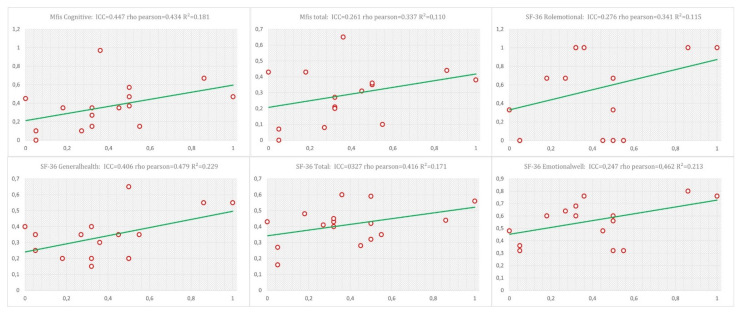
Correlation graphical representation of the higher correlations between the distance assessed by the app with MFIS and SF-36 variables. ICC—intraclass correlation coefficient; MFIS—Modified Fatigue Impact Scale; SF-36—Short Form-36 Health Survey.

Distance evaluated with the RUNZI^®^ app showed an excellent correlation with the 6MWT assessed conventionally (*p* < 0.0001; r = 0.851). No statistical correlations were obtained between the distance assessed by the RUNZI^®^ app with MFIS and the SF-36 (Table 2). However, in Figure 2, higher correlations between the app, MFIS, and SF-36 variables are shown.

Test–retest reliability was found to be close to statistical significance (*p* = 0.058) for distance (m) measured with RUNZI^®^ with an ICC of 0.482. The SEM, MDC, and MDC% values are shown in Table 3.

## 4. Discussion

The objective of this paper was to study the construct validity and test–retest reliability of the RUNZI^®^ app to evaluate aerobic capacity and endurance through the 6MWT in post-COVID-19 syndrome patients. Our results show a significant correlation between 6MWT assessed conventionally and with the free mobile application, indicating its construct validity in post-COVID-19 syndrome patients. This correlation coefficient was higher than 0.80, so interpreted as excellent. However, no correlations were found between fatigue and health-related quality of life. Further, values for the test–retest reliability for the free mobile application were very close to statistical signification.

To the best of our knowledge, this is the first study that has used a free app to assess aerobic capacity and endurance in post-COVID-19 syndrome patients. Prior studies have tried to utilize the 6MWT with mobile applications in healthy subjects or in populations with specific disorders. Smith-Turchyn et al. [22] studied the usability of the *EasyMeasure app* to self-administer the 6MWT in a healthy, young population. They also studied the reliability and construct validity of the app. They found good usability and interesting psychometric properties of the *EasyMeasure app* to measure functional capacity for community-based exercise screening and patient monitoring. Similarly, Jesus et al. [23] assessed the reliability and reproducibility of a self-administered 6MWT in 93 asymptomatic adults using a free smartphone app in indoor and outdoor settings. The self-administered 6MWT had excellent reliability and reproducibility in asymptomatic adults.

Our results coincide with Smith-Turchyn et al. [22] and Jesus et al. [23] in demonstrating the construct validity of the RUNZI app with the 6MWT in an indoor setting. However, our findings could not be extrapolated to other settings as our procedure was conducted in a clinical setting on post-COVID-19 syndrome patients. Salvi et al. [24] studied the accuracy of a mobile application, technically described and developed by Salvi et al. [25], in assessing the 6MWT in an indoor clinical setting, compared to in an outdoor clinical setting in people with pulmonary arterial hypertension. Results concluded the mobile application was a valid tool, with an excellent test–retest reliability of outdoor 6MWT. In our study, test–retest reliability was close to statistical signification with an ICC considered as poor. This finding could be due to several reasons. It is possible our indoor procedure may have impacted the test–retest reliability of the application. Future studies to assess a 6MWT using RUNZI^®^ in an outdoor clinical setting will require a larger sample size of post-COVID-19 syndrome patients. Additionally, the geolocation of the mobile device may have been skewed for a variety of reasons, such as a phone having a poor connection indoors. Our results were obtained under a supervised 6MWT using the RUNZI^®^ app. If future studies wish to use a self-administered 6MWT, then an analysis of the psychometric properties of a self-administered 6MWT using RUNZI^®^ in post-COVID-19 syndrome patients must be completed.

6MWT has also been assessed in other pathological conditions with mobile applications. Ata et al. [26] used the *VascTrac iPhone app* to evaluate the 6MWT, compared to conventionally measured, in 114 people with peripheral artery disease. Their results showed that the app was unable to accurately measure distance. Ziegl et al. [27] used a 6MWT app with heart-failure patients and found reliable and accurate results. Rens et al. [28] used the *VascTrac research app* to assess 6MWT in home-based and clinical settings in people with cardiovascular disease (vascular or cardiac procedures). Their findings suggested that frailty and functional capacity in this population could be monitored remotely in patients with cardiovascular disease, enabling safer and higher-resolution monitoring of patients. Brooks et al. [29] used the *SA-6MWTapp* for independent use at home in people with congestive heart failure and pulmonary hypertension. Distances estimated with the *SA-6MWTapp* during home 6MWTs were highly repeatable and correlated with in-clinic-measured distance.

Our results did not show a significant correlation between the 6MWT assessed by the app, and the MFIS and SF-36. The absence of a correlation between these constructs could be due to several aspects: firstly, the small sample size; secondly, the heterogeneous nature of the clinical symptoms present in patients with persistent COVID. Finally, MFIS and SF-36 are multidimensional scales that refer to the level of fatigue and health-related quality of life in the last two weeks; yet the aerobic capacity was evaluated only on the day they were cited for the study. All these reasons could have had an influence on the absence of correlations between these constructs.

The association between aerobic capacity evaluated by a mobile application and functional capacity has been previously evaluated in other pathological situations [24,26,27,28,29], but this is the first research that has studied the relationship between 6MWT and post-COVID-19 syndrome patients. Fatigue and dyspnea were the most prevalent post-COVID-19 symptoms. In 70% of hospitalized COVID-19 survivors, fatigue was still present 7 months after discharge and 45% of post-COVID-19 syndrome patients exhibited limitations in daily living activities [30,31,32,33]. It is important to support the objective of this study and valid assessment tools for monitoring the functional capacity of these populations by rehabilitation professionals.

Finally, future studies could be conducted in order to analyze other information provided by the RUNZI^®^ app, such as walking speed or cadence, and to study their relationship with traditional 6MWT and other constructs in post-COVID-19 syndrome patients. Previous studies on this subject have been conducted by Proessl et al. [34] and Capela et al. [35] in healthy participants. Future research could use this information in order to determine rehabilitation effects or monitor the progression of other variables offered by this free mobile application in post-COVID-19 syndrome patients.

This study has potential limitations. We used a small sample size, which hampers the detection of statistically significant differences, although these may well exist. Also, a control group was not included. Further, our procedure was conducted under an indoor and supervised protocol, so we could not extrapolate our findings to other settings and conditions. Finally, a non-blinded investigator performed all assessments.

## 5. Conclusions

Instrumental 6MWT assessed by the RUNZI^®^ app for Android^®^ operating system showed an excellent correlation with conventional 6MWT, indicating its construct validity in post-COVID-19 syndrome patients. Further, values for the test–retest reliability for the free mobile application were close to statistical signification with a reliability considered poor in an indoor setting. These findings highlight specific opportunities for a free smartphone-mobile application to serve as an objective assessment of aerobic capacity and endurance in post-COVID-19 syndrome patients.

## Figures and Tables

**Table 1:** Sociodemographic and clinical characteristics of the participants.

Variable	Data (Mean ± SD)
Age	46.06 ± 7.92
Weight (kg)	70.15 ± 20.69
Height (cm)	1.63 ± 0.05
Body Mass Index	26.18 ± 7.06
Length of the lower limbs	84.88 ± 4.58
Mean distance (m) assessed by 6MWT	593.13 ± 89.75
Mean distance (m) assessed by RUNZI^®^ app—First assessment	581.88 ± 57.29
Mean distance (m) assessed by RUNZI^®^ app—Second assessment	585.63 ± 60.21
Physical MFIS Score	28.63 ± 5.32
Cognitive MFIS Score	25.44 ± 9.9
Psychosocial MFIS Score	5.63 ± 1.63
TOTAL MFIS Score	59.69 ± 14.38
SF-36 Physical functioning	55 ± 19.24
SF-36 Role physical	6.25 ± 17.08
SF-36 Role emotional	54.17 ± 43.67
SF-36 Vitality	25.63 ± 14.24
SF-36 Role emotional	56 ± 16.4
SF-36 Social functioning	40.31 ± 19.43
SF-36 Bodily pain	30.78 ± 20.06
SF-36 General health	34.06 ± 14.52
TOTAL SF-36	41.14 ± 11.95

6MWT—6-Minute Walking Test; MFIS—Modified Fatigue Impact Scale; SF-36—Short Form-36 Health Survey.

**Table 2:** Construct validity of RUNZI^®^ app with MFIS and the SF-36.

Measures	Rho Pearson	ICC	*p*-Value
TOTAL MFIS Score	0.26	0.34	0.201
Physical MFIS Score	0.00	0.02	0.953
Cognitive MFIS Score	0.45	0.43	0.093
Psychosocial MFIS Score	0.26	0.29	0.276
SF-36 Physical functioning	0.18	0.31	0.251
SF-36 Role physical	0.00	0.06	0.823
SF-36 Role emotional	0.28	0.34	0.196
SF-36 Vitality	0.14	0.04	0.587
SF-36 Role emotional	0.25	0.46	0.072
SF-36 Social functioning	0.00	0.24	0.366
SF-36 Bodily pain	0.16	0.18	0.516
SF-36 General health	0.41	0.48	0.060
TOTAL SF-36	0.33	0.42	0.109

MFIS: Modified Fatigue Impact Scale; SF-36: Short Form-36 Health Survey.

**Table 3:** Test–retest reliability.

Variable	n	Mean	SD	SEM	MDC	ICC	CI95%	*p*
App 1	16	581.87	57.296	14.324	0.84		551.3 to 612.4	
App 2	16	585.62	60.218	15.054	0.99		553.5 to 617.7	
Combined	32	583.75	57.851	10.226		0.482	562.8 to 604.6	0.058
Difference		−3.75		20.780			−46.1 to 38.6	

SD—standard deviation; SEM—Standard error of measurement. MDC—minimal detectable change; ICC—intraclass correlation coefficient; CI—confidence interval.

## Data Availability

No new data were created or analyzed in this study. Data sharing is not applicable to this article.
